# Current status and controversies in the treatment of neonatal hypoxic-ischemic encephalopathy: A review

**DOI:** 10.1097/MD.0000000000038993

**Published:** 2024-08-02

**Authors:** Hanhong Gao, Hong Jiang

**Affiliations:** a Yan’an University, Shaanxi, Yan’an, China; b Department of Neonatology, Yanan University Affiliated Hospital, Shaanxi, Yan’an, China.

**Keywords:** hypoxic-ischemic encephalopathy, neonatal, treatment

## Abstract

Neonatal hypoxic-ischemic encephalopathy is a type of traumatic brain injury caused by insufficient cerebral perfusion and oxygen supply in the perinatal neonate, which can be accompanied by different types of long-term neurodevelopmental sequelae, such as cerebral palsy, learning disabilities, mental retardation and epilepsy It is one of the main causes of neonatal death and disability, and it has caused a great burden on families and society. Therefore, this article mainly reviews the latest developments in mild hypothermia therapy and related drugs for neonatal hypoxic-ischemic encephalopathy.

## 1. Introduction

Neonatal hypoxic ischemic encephalopathy (HIE) is a type of craniocerebral injury caused by inadequate cerebral perfusion and oxygen supply in perinatal newborns. According to the literature,^[1]^ the prevalence of HIE varies globally, ranging from 3.5/1000 in high-income countries to as high as 26/1000 in low-income countries. Approximately 25% to 30% of these children with HIE may have different types of long-term neurodevelopmental sequelae, such as cerebral palsy, learning disabilities, and seizures.^[2]^ This is one of the major causes of neonatal death and disability and imposes a great burden on families and society.^[3,4]^ To date, due to the complex mechanism of brain injury, no effective treatment has been developed that can completely protect the nerves.^[5]^ Currently, subcooling therapy is considered the only definitive treatment for neonatal HIE, which can significantly reduce the mortality and disability of children with moderate-to-severe HIE and help to improve the prognosis of children with HIE,^[6]^ which helps to improve the prognosis of children with HIE. However, 48% of neonates treated with hypothermia still experience death or varying degrees of neurological damage.^[7]^ Therefore, there is an urgent need to explore more effective treatment strategies. In recent years, domestic and foreign scholars have done a lot of research on neuroprotective drugs, including the combination of various therapies, and now we would like to review the progress of subcooling therapy and drugs for neonatal HIE.

## 2. Subcooling

Subcooling is considered the most effective modality available for the treatment of moderate-to-severe neonatal HIE, with a protective effect on brain tissue by reducing metabolism, inflammatory response, excitability, oxidative damage, and apoptosis in the brain.^[8]^ Weng et al^[9]^ conducted a retrospective study in which 61 cases of neonates with moderate-to-severe HIE were collected and categorized into a therapeutic hypothermia group (n = 36) and a conventional treatment group (n = 25), with the final conclusions suggesting that subcooling for the treatment of moderate-to-severe neonatal HIE improves neurodevelopmental outcomes in infants 0 to 18 months of age and plays a dominant role. There are 2 common clinical modalities of subcooling: (1) selective head hypothermia and (2) whole body hypothermia; Atici et al^[10]^ found that there was no significant difference in adverse effects and short-term outcomes of neonatal HIE between selective head hypothermia and systemic hypothermia treatment modalities. Similarly, a study with a randomized controlled trial on the use of subcooling treatment modalities stated that^[11]^ both selective head hypothermia and systemic hypothermia are effective in reducing infant mortality. However, some studies have concluded that, due to the obvious temperature difference between the surface cortex and the deep brain tissue, selective head hypothermia can only reduce the temperature of the superficial cortical layer, but not that of the deep brain tissue, so it is not yet clear whether selective head hypothermia can achieve the same protective effect as systemic hypothermia. In addition, some studies have found^[12]^ the combination of head cooling and whole-body cooling, in which the brain temperature is controlled by a hypothermia cap and the whole-body temperature is controlled by far-infrared radiation, reduces the temperature of the deep brain tissues without causing the whole-body temperature to be too low. It produces better clinical results and fewer side effects than the 2 classical hypothermia treatments. Therefore, there is still a great deal of controversy about which subcooling treatment to use. In addition, most neonatal HIE is found in regional non-tertiary care facilities, so it is worthwhile to explore how to implement effective hypothermia measures during transportation of the child to a tertiary care facility, and the majority of children are initiated with passive hypothermia treatment at the referring hospital, whereas active hypothermia is usually initiated during transport. A prospective cohort study,^[8]^ evaluated the efficacy and safety of 3 methods of cooling during transportation of 186 newborns with HIE, in which 47 children were given passive cooling, 36 children were given active cooling gel packs, and 103 children were given servo-controlled cooling devices, and ultimately found that there was no significant difference in the adverse events of the 3 modes of transportation cooling. It is worth mentioning that the servo-controlled cooling treatment could maintain the target temperature without significant fluctuations and within the target range when arriving at a tertiary care facility, while reducing hypothermia, so servo-controlled cooling therapy may be the preferred method of HIE neonates during transport.^[13]^ A future area of focus should be long-term neurodevelopmental outcomes following servo-controlled active cooling.

The start time of subcooling treatment for neonatal HIE is within 6 hours after brain tissue hypoxia and ischemia, and it is generally accepted in current research that there are 2 stages of energy failure in the development of neonatal HIE.^[14–16]^ The first stage is the reduction of blood flow and oxygen supply to the brain tissue, which causes a decrease in adenosine triphosphate, followed by Na+-K+-ATP pump failure, depolarization of neuronal cells, intracellular water and sodium retention, cellular edema, ischemia, and ultimately, neuronal cell death. The second stage is the secondary energy failure caused by early excessive energy expenditure a few hours after the occurrence of moderate and severe injury, in which a cascade response of ischemic injury to nerve cells can be triggered, and continuous ischemia produces the excitatory neurotransmitter glutamate, which in turn stimulates calcium influx through activation of the N-methyl-D-aspartate receptor, and calcium influx aggravates the neuronal injury. This ultimately leads to cell death. The “latency period” between the 2 energy depletions is the so-called therapeutic “time window” in which neuroprotective measures to mitigate brain damage can be successfully applied, which is typically 6 to 8 hours.^[17]^ The time window is usually 6 to 8 hours, which means that it is most appropriate to start subcooling within 6 hours after birth in children with moderate-to-severe ischemic-hypoxic encephalopathy. In general, the greater the severity of the hypoxic-ischemic encephalopathy, the longer the duration of subcooling therapy, but the duration of therapy is recommended to be no more than 72 hours.^[18]^ However, it is recommended that the duration of treatment should not exceed 72 hours. This suggests that subcooling treatment lasting 72 hours is more effective than 48 hours or 120 hours.^[19]^ The target temperature for subcooling therapy is generally 2 to 4 °C below brain body temperature, with body surface temperature maintained at 33.5 to 34.0 °C.^[20]^ Shankaran et al^[21]^ did extensive research on moderate-to-severe neonatal HIE, exploring whether 120 hours of subcooling or a body surface temperature of 32.0 °C could reduce mortality or disability in 18-month-old infants, and ultimately concluded that subcooling for more than 72 hours or a body surface temperature as low as 33.5 °C did not further improve the mortality or disability rates of the infants. The above study demonstrates that subcooling treatment for neonatal HIE is not as good as longer duration and lower temperature. Although subcooling therapy can improve the prognosis, there are also many adverse reactions.^[22]^ The most common ones are bradycardia, hypotension, thrombocytopenia, hypoglycemia, and so on. Therefore, multi-center RCTs should be conducted in the future to provide more evidence for clinical implementation.

## 3. Medication

### 3.1. Erythropoietin

Erythropoietin (EPO), a cytokine with neuroprotective and neurocellular regenerative effects, has been used as a potential adjunctive therapy for neonatal ischemic-hypoxic encephalopathy. EPO is synthesized in the liver during the fetal period and in the liver and kidney during the neonatal period, and has anti-inflammatory, antiapoptotic, antioxidant, and neural cell regenerative properties.^[23–26]^ Currently, EPO treatment has been shown to be neuroprotective and neurorestorative in neonatal HIE, where astrocytic EPO has been recognized as a key mediator of neuroprotection under hypoxic conditions.^[27]^ Studies^[28,29]^ have shown that EPO treatment of moderate-to-severe neonatal hypoxic-ischemic encephalopathy reduces the incidence of brain damage, cerebral palsy, and cognitive dysfunction. However, treatment with EPO alone, while not increasing the risk of adverse events, did not improve neurologic injury or reduce mortality.^[30]^ Further consideration of EPO in combination with adjuvant therapy is therefore warranted. A recent report of a phase II clinical trial in neonates with HIE showed that,^[31]^ better neuronal recovery at 12 months in children treated with EPO in combination with subcooling compared with subcooling alone. Thus, the efficacy of subcooling in combination with EPO for the treatment of neonatal HIE looks promising at this time. The therapeutic window for EPO is typically 6 to 12 hours or longer. It is also currently controversial how much dose of rEPO combined with subcooling can further improve prognosis. In a randomized controlled trial in term infants with HIE, repeated administration of high doses of rEPO (1000–2500 IU/kg) was found to be neuroprotective. Prophylactic treatment with high doses of rEPO (1000–3000 IU/kg) has also been shown to improve neurodevelopmental outcomes in studies of preterm infants. In addition, in a phase I/II trial in newborns with HIE,^[32]^ using a therapeutic measure of subcooling therapy combined with EPO (1000 U/kg), the children demonstrated good tolerance and produced neuroprotective plasma concentrations in vivo. At the same time, a study^[33]^ found that subcooling in combination with EPO in rat pups with focal vascular brain injury showed that this neuroprotective effect was more frequent in females, and the explanation for this difference may include gender regulation of responsiveness to R-HU-EPO in the kidney and a significantly higher frequency of EPO receptor alleles in females than in males. In summary, a large number of clinical trials are needed to determine the optimal dose, onset, and duration of EPO treatment, taking into account the severity of brain injury and gender.

### 3.2. Melatonin

Melatonin is an endogenous indoleamine secreted by the pineal gland, and other sources can be found in the retina, bone marrow cells, platelets, skin, lymphocytes, hard glands, cerebellum, and especially in the gastrointestinal tract of vertebrates, but recent studies have shown that melatonin is mainly produced in the mitochondria, and that melatonin’s main functions include regulating circadian patterns, such as fluctuations in body temperature and sleep cycles, seasonal reproduction, boosting the immune system and regulating glucose levels.^[34]^ Melatonin also has multiple hormonal functions. Melatonin also has multiple hormonal functions with paracrine and autocrine effects, and has antioxidant, antiapoptotic, and anti-inflammatory properties.^[3]^ Melatonin is also known to have multiple hormonal functions, paracrine and autocrine, with antioxidant, antiapoptotic, and anti-inflammatory properties. Over the past 20 years, several studies^[35,36]^ have demonstrated the protective effects of melatonin in experimental models of perinatal asphyxia and clinical perinatal asphyxia, and relevant animal experiments have found that^[37]^ subcooling combined with melatonin exerts a better neuroprotective effect than either alone in the treatment of brain injury in newborn lambs. Although melatonin has potential neuroprotective effects, its role in hypoxic-ischemic brain injury and its underlying mechanisms remain unknown. Gou et al^[38]^ explored the role and mechanism of melatonin in a rat model of hypoxic-ischemic brain injury. Ultimately, they found that exogenous melatonin treatment ameliorated the pathological changes induced by hypoxic-ischemic encephalopathy, inhibited neuronal death, and promoted hippocampal neuronal survival. Qin et al reported^[39]^ that melatonin could inhibit the overactivity of NLRP3 inflammasome by enhancing mitochondrial autophagy and suppressing TLR4/NF-κB pathway activity. This suggests that melatonin may be a promising therapeutic approach to attenuate massive neuronal cell death induced by endotoxin and ischemic hypoxia in neonatal rats. Current studies have only confirmed the short-term therapeutic effects of melatonin, with Berger et al^[40]^ finding that 3 injections of 10 mg/kg of melatonin within the first 25 hours of melatonin treatment in hypoxic-ischemic neonatal rats produced only a transient and small neuroprotective effect, which may not be sufficient to attenuate the development of long-term brain damage after hypoxia-ischemia. Despite strong experimental data supporting the role of melatonin as a neuroprotective agent in hypoxic-ischemic encephalopathy (alone and as an adjunct to hypothermia), relevant clinical trials with adequate neonatal sample sizes are lacking, and large-sample safety and efficacy multicenter randomized controlled trials are urgently needed to confirm the neuroprotective effects of melatonin in neonatal HIE.

### 3.3. Xenon

Xenon, an inhalable anesthetic inert gas for adults, acts as a potent inhibitor of the N-methyl-D-aspartate subtype of glutamate receptor, leading not only to a decrease in excitatory neurotransmitter release, a key step in the neurotoxic cascade of neonatal HIE. It also inhibits other subtypes of glutamate receptor subtypes, that is, AMPA and Kainate receptors.^[41]^ In addition, xenon modulates inflammatory cytokines such as tumor necrosis factor alpha and interleukin 6, thus suggesting that xenon may offer new therapeutic approaches for neurological injuries such as ischemic and traumatic brain injuries, Sabir et al^[42]^ found that breathing the inert gas xenon enhanced hypothermic neuroprotection after hypoxia-ischemia in small and large model neonates. Although the underlying mechanisms of enhancement are not fully understood, the combined effects of xenon and subcooling may be synergistic, and several studies^[43,44]^ have demonstrated that xenon enhances the neuroprotective effects of subcooling treatment when administered at 50% concentration within 5 hours of hypoxia in neonates. In a feasibility study in human neonates, researchers found that xenon enhanced the neuroprotective effects of subcooling at 72 hours.^[45]^ Researchers found that 72 hours of hypothermia combined with 18 hours of 50% xenon maximized neuroprotection. Similarly, Dingley et al^[46]^ demonstrated that inhalation of 50% xenon for up to 18 hours combined with 72 hours of subcooling was feasible in children with neonatal encephalopathy, and no adverse effects were observed at 18 months of follow-up. The benefits of xenon alone or in combination with subcooling for neonatal HIE have been demonstrated in multiple in vitro and in vivo studies, and with more research from national and international scholars, it may only be a matter of time before xenon and subcooling become the standard of care for neonatal HIE. The use of xenon is promising, but further clinical studies are needed to confirm the feasibility of its routine use, as well as the optimal timing, concentration, and duration for use in human neonates with hypoxic-ischemic conditions.

### 3.4. Magnesium sulfate

Magnesium sulfate prevents calcium entry into cells by noncompetitive voltage-dependent inhibition of NMDA-type glutamate receptors. A study^[47]^ reported that magnesium sulfate is neuroprotective against brain damage by preventing this excitotoxic effect in nerve cells. In addition, magnesium sulfate inhibits the reduction of secondary inflammation-induced injury through cell membrane stabilization and free radical formation.^[48,49]^ Although studies have shown that prenatal administration of magnesium sulfate to pregnant women reduces the risk of cerebral palsy in preterm infants, it has not been used routinely for neuroprotection. Magnesium sulfate has been used clinically as a cytoprotective agent for neonatal HIE brain injury in some countries, including Japan. However, it is unclear how magnesium sulfate exerts this effect and how it acts on the various types of cells within the neonatal brain. Seyama et al found that^[50]^ that pretreatment with magnesium sulfate reduced oligodendrocyte pre-cell death, thereby attenuating cerebral white matter injury. Similarly, Imamoğlu et al^[51]^ evaluated the effects of systemic magnesium sulfate on the retina of a rat model of preterm hypoxia-ischemia and concluded that pretreatment and treatment with magnesium sulfate in a rat model of preterm hypoxia-ischemia may reduce neuronal apoptosis. Magnesium sulfate combined with subcooling and EPO has synergistic neuroprotective effects in the treatment of neonatal HIE. A study^[52]^ reported that for neonatal HIE that met the criteria for subconditioning, EPO 300 U/kg administered every other day for 2 weeks, 250 mg/kg magnesium sulfate for 3 days, and subconditioning were initiated within 6 hours of birth, and the final results found that this therapeutic strategy was feasible for patients with neonatal HIE. In addition, the combination of magnesium sulfate and melatonin can also effectively improve the clinical symptoms of hypoxic-ischemic encephalopathy, shorten the course of the disease, and promote the recovery of neurological function, and it is safe. In a randomized controlled trial of neonates with moderate HIE (Sarnat II), children were randomly divided into 2 groups,^[53]^ with the first group receiving magnesium sulfate and melatonin and the second group receiving melatonin only. Serum S100-B concentrations were measured at baseline, day 2, and day 6, and were found to be lower in group 1 on days 2 and 6 compared with baseline. However, S100-B in group 2 started to decrease only on day 6. The results suggest that magnesium sulfate combined with melatonin treatment may also reduce brain damage in neonatal HIE. The above indicates that magnesium sulfate can improve neonatal HIE brain injury in the short term without obvious side effects, and further experiments are needed in the later stage to determine how the long-term efficacy of magnesium sulfate is, and there is a trend of relative increase in mortality rate of magnesium sulfate treatment, which should be closely monitored in future experiments.

### 3.5. Topiramate

Topiramate is an antiepileptic drug with AMPA/alginate receptor antagonism, which has been clinically used for the treatment of refractory neonatal seizures. In recent years, a large number of scholars at home and abroad have begun to focus their attention on the study of the mechanism of neuroprotective aspects of topiramate, which prevents the sodium channel, high-pressure activation of calcium currents, carbonic anhydrase isoforms, the transition pore of mitochondrial permeability, and, finally, by increasing the pre-glial cell survival, decreasing mitochondrial dysfunction and neuronal apoptosis, as well as decreasing seizure activity, thus exhibiting its neuroprotective effects.^[41,54]^ Topiramate has been shown to be effective in reducing brain damage, and Nuñez-Ramiro et al^[55]^ conducted a randomized controlled double-blind topiramate/placebo multicenter trial on this topic, concluding that topiramate is neuroprotective and can be safely used in the neonatal period. The combination of topiramate and subcooling enhances neuroprotection in neonatal HIE and is effective in controlling neurobehavioral conditions such as seizures. Recent studies have found that^[56,57]^ topiramate (10 mg/kg/day) for 3 days during subconditioning in moderate-to-severe neonatal HIE did not reduce mortality or improve neurologic sequelae at 18 to 24 months, but it did substantially reduce seizure rates. The safe dose of topiramate in neonates with HIE is 10 mg/kg, and the study^[58]^ found that increasing the dose to 25 mg/kg/day did not increase the efficacy of topiramate. This suggests that short-term moderate doses of TPM are protective against hypoxic-ischemic brain damage, but long-term or excessive use may cause new damage to the brain and reduce cognitive performance. In conclusion, although a large number of animal experiments have confirmed the neuroprotective effect of topiramate, the clinical use of topiramate has not yet been fully matured, and it is still necessary to focus on the safe dosage of topiramate in children with HIE.

### 3.6. Other

Molecular hydrogen reduces the levels of peroxynitrite anions and hydroxyl radicals in ischemic stroke, and in particular its antioxidant and anti-inflammatory effects have attracted much attention.^[59]^ In studies of adult diseases, hydrogen has been shown to be neuroprotective in diseases such as cerebral ischemia and traumatic brain injury, as well as in neurodegenerative diseases such as Alzheimer disease. Animal and human studies have validated the safety and feasibility of molecular hydrogen. Nakamura et al^[60]^ evaluated short-term neurological outcomes and histological findings in 5-day-old piglets with hypoxic-ischemic encephalopathy, and the results reported that hydrogen therapy combined with subcooling has neuroprotective potential. However, the effects of hydrogen on cerebral circulation and oxygen metabolism as well as prognosis are unknown. Despite extensive research on its efficacy in adults, only a few studies have explored its use in pediatrics and neonatal medicine. Htun et al^[61]^ did a retrospective study and found that hydrogen as a single agent or in combination with therapeutic hypothermia exhibited short- and long-term neuroprotective effects in a neonatal HIE model. Future studies should focus on the different routes of administration of hydrogen as well as the effects on brain damage and the main mechanisms of hydrogen neuroprotection. Mesenchymal stem cells (MSCs) have potent anti-inflammatory and antiapoptotic properties, especially the pleiotropic neuroprotection of MSCs,^[62,63]^ an experimental animal study found^[64]^ that injection of human embryonic stem cell-derived MSCs into a mouse model resulted in some improvement in neurobehavior of the treated mice. Devyaltovskaya et al^[65]^ reported 2 clinical cases of application of umbilical cord-derived somatic MSCs for the treatment of hypoxic-ischemic encephalopathy rehabilitation in very preterm infants (27–28 weeks of gestation, weighing 900 and 870 g, respectively), in which children were sampled at birth with umbilical cord-derived MSCs, and the cells were injected at doses of 1.6 to 7 million per kilogram (MW/kg) at the ages of 3, 6, 12 months (the first patient) and 3, 6, 9, 15 months (the second patient). 7 million/kg of cells were injected. The final results showed that the cell therapy had no significant adverse effects and favored neuromotor development in children. This suggests that MSC transplantation will become a new treatment modality in the future, but the route of application of MSC, the optimal timing, and the route and dose of administration are still unknown. Insulin-like growth factor-1 is one of the major factors regulating cell survival, proliferation and maturation and contributes to interneuron development. Vaes et al^[66]^ studied 2 experimental models, intranasal MSCs or insulin-like growth factor I and showed that both modalities were able to restore the majority of neuronal cells in brain-injured mice, and that the impaired social behavior was partially restored. Allopurinol, a xanthine oxidase inhibitor, is neuroprotective by reducing oxygen radical production and brain damage in experimental, animal and early human ischemia and reperfusion studies. An animal model study found^[67]^ that protein oxidation and lipid peroxidation were significantly reduced when subcooling was combined with allopurinol treatment, thereby enhancing the neuroprotective effects of HIE. Similarly, a double-blind, placebo-controlled study (ALBINO, NCT03162653),^[58]^ is currently investigating the neuroprotective effects of allopurinol in neonates with HIE. It is hoped that more experimental studies and clinical data will be available in the future to support its efficacy. N-Acetylcysteine (NAC) is a compound with antioxidant properties that exerts a potential neuroprotective effect in a variety of neurological disorders, including cerebral ischemia-induced injury.^[68]^ Moreover, NAC and vitamin D provide effective neuroprotection in animal models of severe or inflammatory sensitization to HIE. Jenkins et al^[69]^ conducted a 10-day NVD (NAC [25, 40 mg/kg q12h] in combination with 1,25(OH)2 D3 [0.05 mg/kg q12h, 0.03 mg/kg q24h]) and concluded that a low, safe dose of NVD administered for the treatment of neonatal HIE reduces oxidative stress in the plasma and central nervous system, improves CNS energy, and may improve neurologic development from 2 to 4 years of age. Therefore, more clinical studies in neonates are warranted to improve the prognostic outcome of neonatal HIE.

## 4. Summary

Currently, there is no effective treatment option for neonatal hypoxic-ischemic encephalopathy, and this paper mainly reviews neuroprotective interventions related to neonatal HIE, because the pathogenesis of neonatal HIE is complex, with different severity and HIE stages and inflammatory states, which may need to be targeted for individualized neuroprotective measures, and future studies should also focus on preventive strategies for neonatal HIE. A large number of animal model studies have confirmed the neuroprotective effects of several interventions in this paper, but their routes of administration, timing of administration, mode of administration, and dosage are still controversial, and more randomized controlled trials with more data are needed in the future to support their efficacy and safety, and we also expect that they can be implemented in the clinic as soon as possible.

## Author contributions

**Supervision:** Hong Jiang.

**Writing – original draft:** Hanhong Gao.

**Writing – review & editing:** Hanhong Gao.
